# Magnetic resonance imaging appearance of breaststroker’s knee

**DOI:** 10.1007/s00247-022-05407-6

**Published:** 2022-06-08

**Authors:** Karen Y. Cheng, Henry Chambers, Jerry R. Dwek

**Affiliations:** 1 University of California, San Diego, San Diego, CA USA; 2 Department of Radiology, Rady Children’s Hospital, 3020 Children’s Way, San Diego, CA 92123 USA

**Keywords:** Breaststroke, Knee, Magnetic resonance imaging, Overuse, Swimming

## Abstract

Breaststroker’s knee is an overuse syndrome resulting from similar repetitive movements in competitive swimmers that has been described in the orthopedic literature. The typical symptoms are medial knee pain with tenderness to palpation at the tibial collateral ligament or inferomedial patella. Despite these localizing symptoms on clinical exam, arthroscopic studies have failed to demonstrate a specific structural abnormality corresponding to this syndrome, although some have reported thickened medial synovial plica, medial-predominant synovitis or patellofemoral cartilage loss in association knee pain with breaststroke swimmers. We present a case of medial knee pain in a young breaststroke swimmer with magnetic resonance imaging (MRI) findings of marrow edema in the anterior aspect of the medial femoral condyle. To our knowledge, this is the first reported case of MRI findings in breaststroker’s knee .

## Introduction

Breaststroker’s knee was first described by Kennedy et al. [1] in 1978. After surveying 2,496 Canadian swimmers, the authors discovered that 236 of the swimmers had orthopedic problems. Of these swimmers with musculoskeletal complaints, 70 reported medial knee pain, and these symptoms were related specifically to the breaststroke [1]. Based on clinical exam findings of point tenderness at the adductor tubercle, they concluded that the most common cause of the symptoms was chronic irritation of the tibial collateral ligament and recommended at least 2 months of rest from training per year to avoid excessive valgus and external rotation stress on the knee. The authors suggested that other possible etiologic factors included: hypermobile or torn medial meniscus, mobile patella with early chondromalacia, repetitive stress of the capsular ligament causing rotatory instability, or osteochondritis dissecans. However, a subsequent study in which nine swimmers with breaststroker’s knee underwent arthroscopic evaluation revealed no such structural abnormalities, only medial synovitis [2]. Here, we present magnetic resonance imaging (MRI) findings of medial femoral condylar marrow edema related to breaststroker’s knee in a teenage boy. Based on our review of the literature, the MRI features of breaststroker’s knee have not previously been reported.

## Case report

**Fig. 1:**
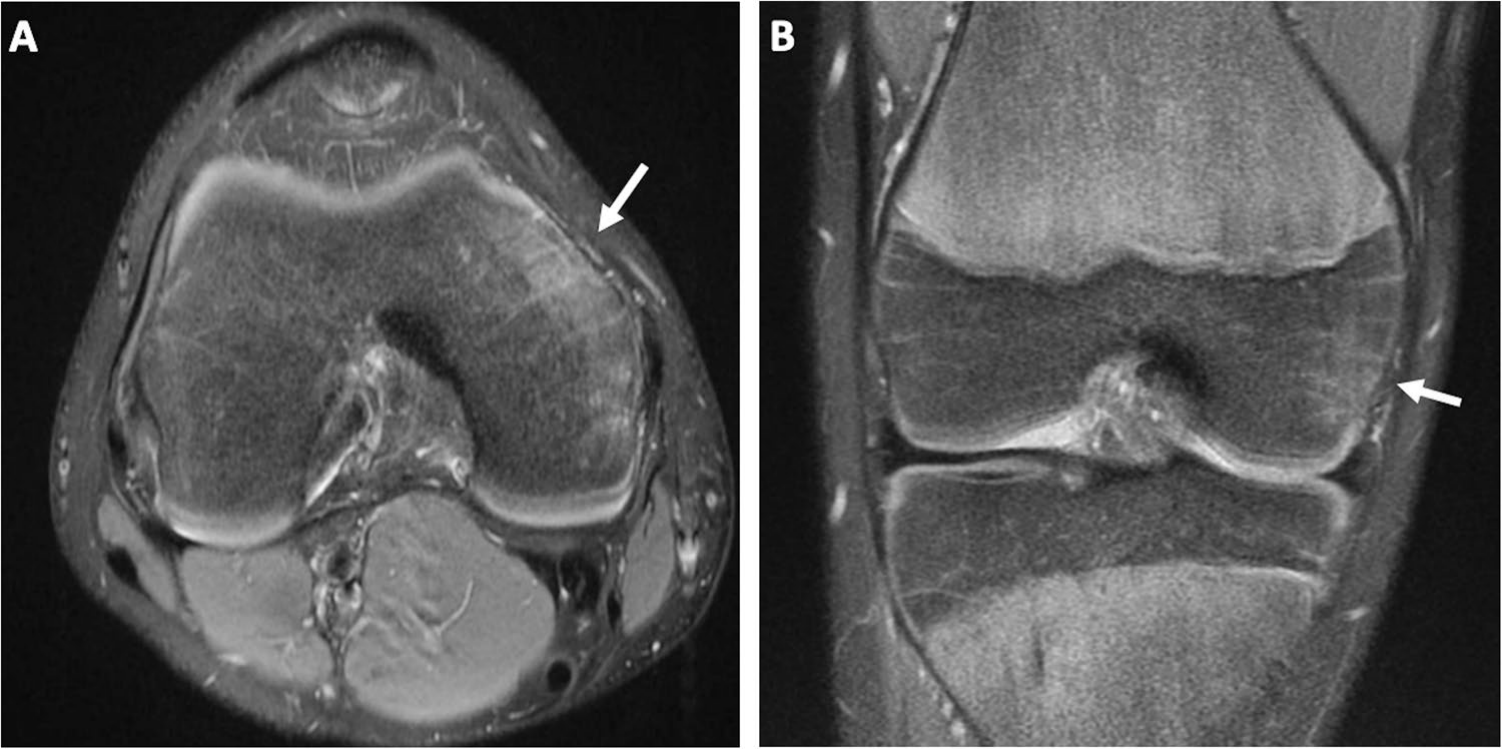
Magnetic resonance imaging of the right knee in a 13-year-old boy. Axial (**a**) and coronal (**b**) proton density fat-saturated images demonstrate marrow edema involving the anterior aspect of the medial femoral condyle (*arrows*)

**Fig. 2:**
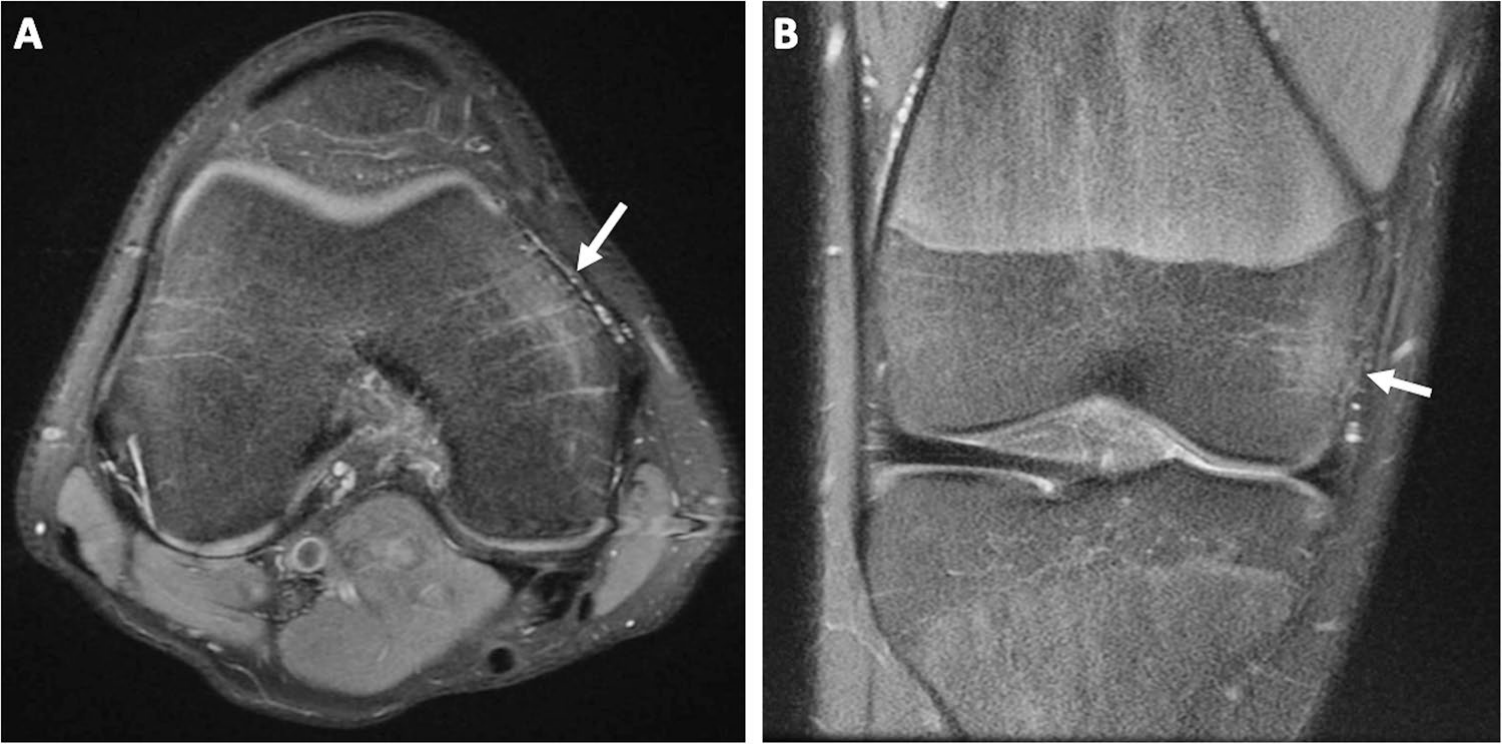
Magnetic resonance imaging of the right knee at 15 years of age. Axial (**a**) and coronal (**b**) proton density fat-saturated sequences again demonstrate marrow edema involving the anterior aspect of the medial femoral condyle (*arrows*)

A 13-year-old boy underwent MRI for knee pain. He had no significant past medical history. The patient did not recall or report any antecedent trauma. Radiographs of the knee were normal. MRI demonstrated marrow edema in the anterior aspect of the medial femoral condyle (Fig. 1) without evidence of ligamentous, capsular, meniscal or chondral pathology. The abnormal signal was anterior to the tibial collateral ligament and lay directly beneath the medial patellar retinaculum and superficial band of the tibial collateral ligament. The retinaculum and ligament themselves were normal and there was no edema in the overlying soft tissues. The possibility of a stress injury was raised at this time, but the physis lacked significant stress changes. Two years later, at age 15, the boy again underwent MRI for knee pain. At this time, he revealed that his symptoms had begun when he first started competitively swimming breaststroke. MRI again showed focal marrow edema involving the anterior aspect of the medial femoral condyle (Fig. 2).

At this time, the medical record contained more history and specifically mentioned that the patient was a competitive swimmer, and the pain began soon after he began to do the breaststroke. A diagnosis of breaststroker’s knee was made. The patient stopped doing the breaststroke primarily and the pain resolved.

## Discussion

In this report, we describe the MRI findings in a patient with a clinical presentation compatible with breaststroker’s knee. Breaststroker’s knee is a common entity among competitive breaststroke swimmers and may produce significant discomfort that prevents athletes from reaching their full training potential. Although there has been some interest in the epidemiology and possible treatment of this syndrome in the orthopedic literature as well as in the lay literature, there have thus far been no descriptions of its imaging characteristics. Based on clinical exam findings, previous authors have attributed the symptoms to pathology of the tibial collateral ligament [1–4], patellofemoral joint friction or chondromalacia [3, 4], and inflammation related to a thickened medial plica [5]. In a small case series of swimmers with breaststroker’s knee evaluated by arthroscopy, no injury of the tibial collateral ligament or other knee ligaments, meniscus or any other structure within the knee was identified [2]. In another study, two patients who had been swimming competitively for more than 8 years underwent arthroscopic evaluation and were found to have patellofemoral osteoarthrosis [3]. Radiographs in breaststroker’s knee are reportedly normal [3].

This patient with medial knee pain had marrow edema in the medial femoral condyle corresponding to the location of his symptoms, which began when he started competitively swimming the breaststroke and were again noted when he underwent repeat imaging for the same symptoms 2 years later. There was no other internal derangement of the knee. No injury to the tibial collateral ligament was identified. There was no evidence of medial synovitis, prominent medial plica or patellofemoral chondromalacia. Kennedy et al. [1] in 1978 used underwater photography to analyze the whip kick used by breaststrokers and found that the knee moves in the later phase from flexion to extension with valgus stress and external rotation applied to the knee. They further studied the stress on the tibial collateral ligament using a strain gauge with fresh cadavers and a simulated whip kick. They found that tension in the tibial collateral ligament increased when the knee moved from flexion to extension, increasing again during valgus stress and ending with a dramatic increase in tension during the final external rotation of tibia on femur. These findings were confirmed by Keskinen et al. [2], who performed a biomechanical study using a transparent pool finding that during certain phases of the whip kick high angular velocities were sustained. Their conclusion was that high angular velocities as well as external rotation of the tibia relative to the femur was the primary cause.

We postulate that the abnormal signal represents a friction syndrome from the overlying tibial collateral ligament.

Breaststroker’s knee is thought to be directly correlated to patient age, years of competitive swimming, training distance and reduced warm-up distance [5]. Recommendations to treat breaststroker’s knee have varied, including resting for a part of the year to prevent excessive abnormal stress [1], year-round training to avoid early-season symptoms [5], changing hip abduction angle at kick initiation [4], decreasing external rotation of the tibia [1], and keeping the legs together during hip and knee flexion and the thighs together during knee and hip extension [3].
